# Tongue fasciculations with denervation pattern in osmotic demyelination syndrome: a case report of diagnostic dilemma

**DOI:** 10.1186/s13104-018-3287-8

**Published:** 2018-03-14

**Authors:** H. M. M. T. B. Herath, S. P. Pahalagamage, Sunethra Senanayake

**Affiliations:** National Hospital, Colombo, Sri Lanka

**Keywords:** Osmotic demyelination syndrome, Central pontine myelinolysis, Hypoglossal nerve denervation, Tongue fasciculations

## Abstract

**Background:**

The pathogenesis of osmotic demyelination syndrome is not completely understood and usually occurs with severe and prolonged hyponatremia, particularly with rapid correction. It can occur even in normonatremic patients, especially who have risk factors like alcoholism, malnutrition and liver disease. Bilateral tongue fasciculations with denervation pattern in electromyogram is a manifestation of damage to the hypoglossal nucleus or hypoglossal nerves. Tongue fasciculations were reported rarely in some cases of osmotic demyelination syndrome, but the exact mechanism is not explained.

**Case presentation:**

A 32-year-old Sri Lankan male, with a history of daily alcohol consumption and binge drinking, presented with progressive difficulty in walking, dysphagia, dysarthria and drooling of saliva and alteration of consciousness. On examination he was akinetic and rigid resembling Parkinsonism with a positive Babinski sign. Clinical features were diagnostic of osmotic demyelination syndrome and MRI showed abnormal signal intensity within the central pons and basal ganglia. He also had tongue fasciculations. The electromyogram showed denervation pattern in the tongue with normal findings in the limbs. Medulla and bilateral hypoglossal nerves were normal in MRI.

**Conclusion:**

We were unable to explain the exact mechanism for the denervation of the tongue, which resulted in fasciculations in this chronic alcoholic patient who developed osmotic demyelination syndrome. The hypoglossal nuclei are located in the dorsal medulla and radiologically undetected myelinolysis of the medulla is a possibility. Hypoglossal nerve damage caused by methanol or other toxic substances that can contaminate regular ethyl alcohol is another possibility, as it is known to cause neurological and radiological features similar to osmotic demyelination syndrome with long-term exposure. So these toxic substances might play a role in chronic alcoholic patients with central pontine myelinolysis.

**Electronic supplementary material:**

The online version of this article (10.1186/s13104-018-3287-8) contains supplementary material, which is available to authorized users.

## Background

Osmotic demyelination syndrome (ODS) or central pontine myelinolysis (CPM) is a complication of severe and prolonged hyponatremia, particularly with rapid correction. It results in non-inflammatory demyelination within the central pons and also in extra-pontine regions, including the mid brain, thalamus, basal nuclei, and cerebellum. The pathogenesis of ODS is not completely understood. During chronic hyponatremia, osmotically active substances and water is lost from the brain cells and these solutes cannot be as quickly replaced with rapid correction of the hyponatremia. These rapid shifts of intracellular, extracellular, intravascular water, sodium, chloride and organic osmolytes produce relative glial dehydration and myelin degradation and/or oligodendroglial apoptosis [1, 2]. Gankam Kengne et al. hypothesised that rapid correction of hyponatremia triggers apoptosis of astrocytes, disruption of the astrocyte-oligodendrocyte network, secondary inflammation with up regulation of inflammatory cytokines genes, microglial activation leading finally to demyelination [3].

Even though it usually occurs after rapid correction of hyponatremia, it can occur even in normonatremic patients, especially who have risk factors like alcoholism, malnutrition, liver disease, liver transplantation, diabetes mellitus, hypokalemia, pituitary surgery, lithium toxicity, hypophosphatemia, chemotherapy and chronic renal failure [4–9]. The clinical manifestations of ODS include various degrees of encephalopathy, behavioral disturbances, lethargy, confusion, disorientation, obtundation, dysarthria and dysphagia [10, 11]. Severely affected patients may develop coma or locked in syndrome [10, 12]. Involvement of corticospinal and corticobulbar tracts within the pons leads to pseudo-bulbar palsy and spastic quadriplegia. Extra-pontine myelinolysis leads to extrapyramidal signs and Parkinson like manifestations [13–15].

The hypoglossal nerve is the twelfth cranial nerve, which innervates the muscles of the tongue. Damage to the hypoglossal nucleus or hypoglossal nerve (the lower motor neuron) results in weakness, atrophy and fasciculation of the tongue, which are signs of a lower motor neuron lesion. Bilateral tongue fasciculations with denervation pattern in electromyogram (EMG) is typically seen in anterior horn cell disease [16], but rarely reported in cases of damage to bilateral hypoglossal nerves along its pathway [17–23]. As mentioned above, ODS/CPM causes pseudo-bulbar palsy with a spastic tongue.

Here we describe a young male with a history of chronic alcoholism, who presented with features of ODS. Even though his sodium was normal, clinical manifestation and magnetic resonance imaging (MRI) findings were typical of ODS. During recovery he developed bilateral tongue fasciculations and MRI did not show any lesion in the medulla or along the hypoglossal pathway. Tongue fasciculations were reported in some cases of CPM rarely, but the exact mechanism was not explained [24].

## Case presentation

A 32-year-old Sri Lankan male, who was living in South Korea for the last 4 years admitted to us with altered level of consciousness, which progressed over 2 weeks. While in Korea, he was consuming alcohol daily (Soju: “Korea’s most popular alcoholic beverage” 150 mL/day) and during weekends he used to binge drink. Following a binge of alcohol he developed anorexia, nausea, lethargy, progressive difficulty in walking, dysphagia, dysarthria and drooling of saliva, which was progressive. Then he came back to Sri Lanka where his family members noticed behavioral changes, reduced speech and progressive alteration of consciousness. There was no preceding history of headache, fever, toxin exposure and he did not have diabetes, hypertension, hypothyroidism, chronic liver cell disease, consumption of caffeine, tobacco or illegal drugs. His nutritional status was adequate and there was no family history of neurological disorders.

On examination, he was an averagely built person who was drowsy, disoriented, akinetic and rigid resembling Parkinsonism. He had reduced speech and drooling of saliva. He did not have ophthalmoplegia and the vision, fundoscopic examination and light reflexes were normal. Rest of the cranial nerve examination was also normal without any subtle cranio-bulbar atrophy. Upper and lower limbs were rigid with increased reflexes and positive Babinski sign. Power was difficult to assess due to rigidity. His sensory examination was normal and did not have muscle atrophy or fasciculations. He was afebrile and there were no signs of meningism or chronic liver cell disease.

**Fig. 1:**
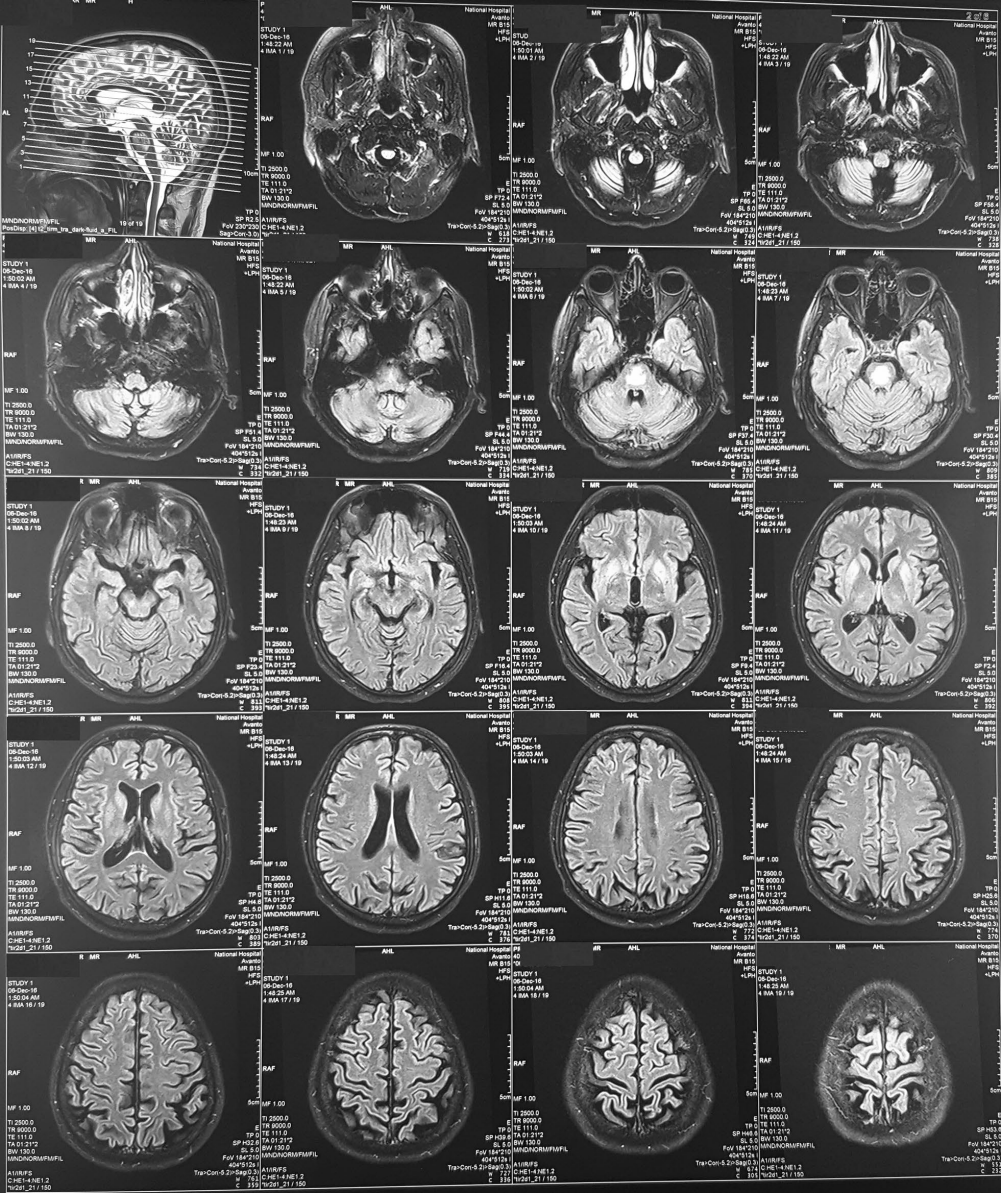
MRI, done 2 weeks after the onset of symptoms, showing high signal intensity within the central pons and bilateral basal ganglia in fluid-attenuated inversion recovery

His basic investigations were normal including serum sodium, liver function tests and inflammatory markers (Table 1). Blood picture showed normal and macrocytic red blood cells and normal neutrophils. Serum B12 level was normal. Electroencephalography demonstrated diffuse bi-hemispheric slowing. MRI, done 2 weeks after the onset of symptoms showed abnormal signal intensity within the central pons with low signal intensity in T1 and high signal intensity in T2 and fluid-attenuated inversion recovery (FLAIR) (Fig. 1). There was no contrast enhancement or diffusion restriction. Pre-pontine cistern appeared normal with no evidence of mass effects. Bilateral basal ganglia also showed symmetrical mild T2 and FLAIR hyper intensity (Fig. 1). These were suggestive of CPM with extra pontine myelinolysis involving basal ganglia. Cerebrospinal fluid (CSF) analysis was normal with no cells, normal protein and sugar levels. CSF was also negative for tuberculosis Polymerase chain reaction, tuberculosis culture and oligoclonal bands.

**Table 1:** Basic investigations

Investigation and value	Normal range	Investigation and value	Normal range
WBC 7.03 × 10^3^/μL	4–10	Neutrophils 5.18 × 10^3^/μL	2–7
Lymphocytes 0.80 × 10^3^/μL	0.8–4	Platelets 204 × 10^3^/μL	150–450
Hemoglobin = 14.6 g/dL	11–16		
MCV 101.2 fL	80–100	MCH 33.0 pg	27–34
MCHC 32.6 g/dL	32–36		
AST 32 U/L	10–35	ALT 28 U/L	10–40
Alkaline phosphatase = 110 U/L	100–360	INR 1.08	
Albumin = 42 g/L	36–50	Globulin 21.0 g/L	22–40
Serum creatinine = 85 μmol/L	60–120	Serum sodium = 138 μmol/L	135–148
Serum potassium = 3.7 μmol/L	3.5–5.1		
C reactive protein = 6 mg/L	0–6	Erythrocyte sedimentation rate = 25 mm in 1 h	
Ionized calcium = 1.11 mmol/L	1.0–1.3	Serum magnesium = 0.85 mmol/L	0.8–1.1
Fasting blood sugar = 100 mg/dl	< 100	HbA1c = 5.4	< 5.5%
TSH = 0.813 μIu/mL	0.5–4.5	Free T4 = 1.76 ng/dL	0.78–2.19 ng/dL

*WBC* white blood cell count; *MCV* mean corpuscular volume; *MCH* mean corpuscular hemoglobin; *MCHC* mean corpuscular hemoglobin concentration; *AST* aspartate transaminase; *ALT* alanine transaminase; *INR* international normalized ratio

**Fig. 2:**
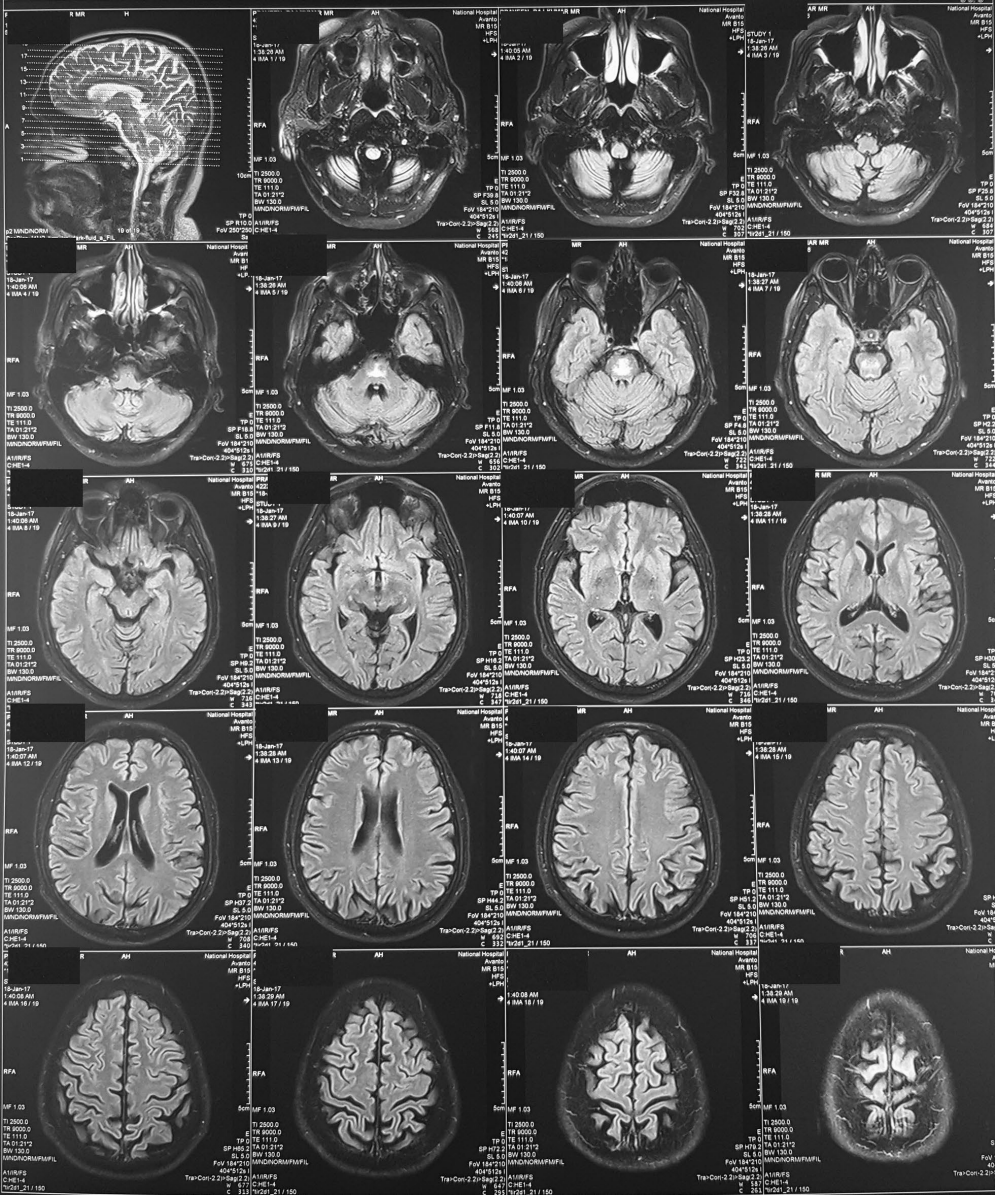
MRI, done 1 month after the first MRI, showing FLAIR high intensity in the central pons with resolution of the changes in the basal ganglia

**Fig. 3:**
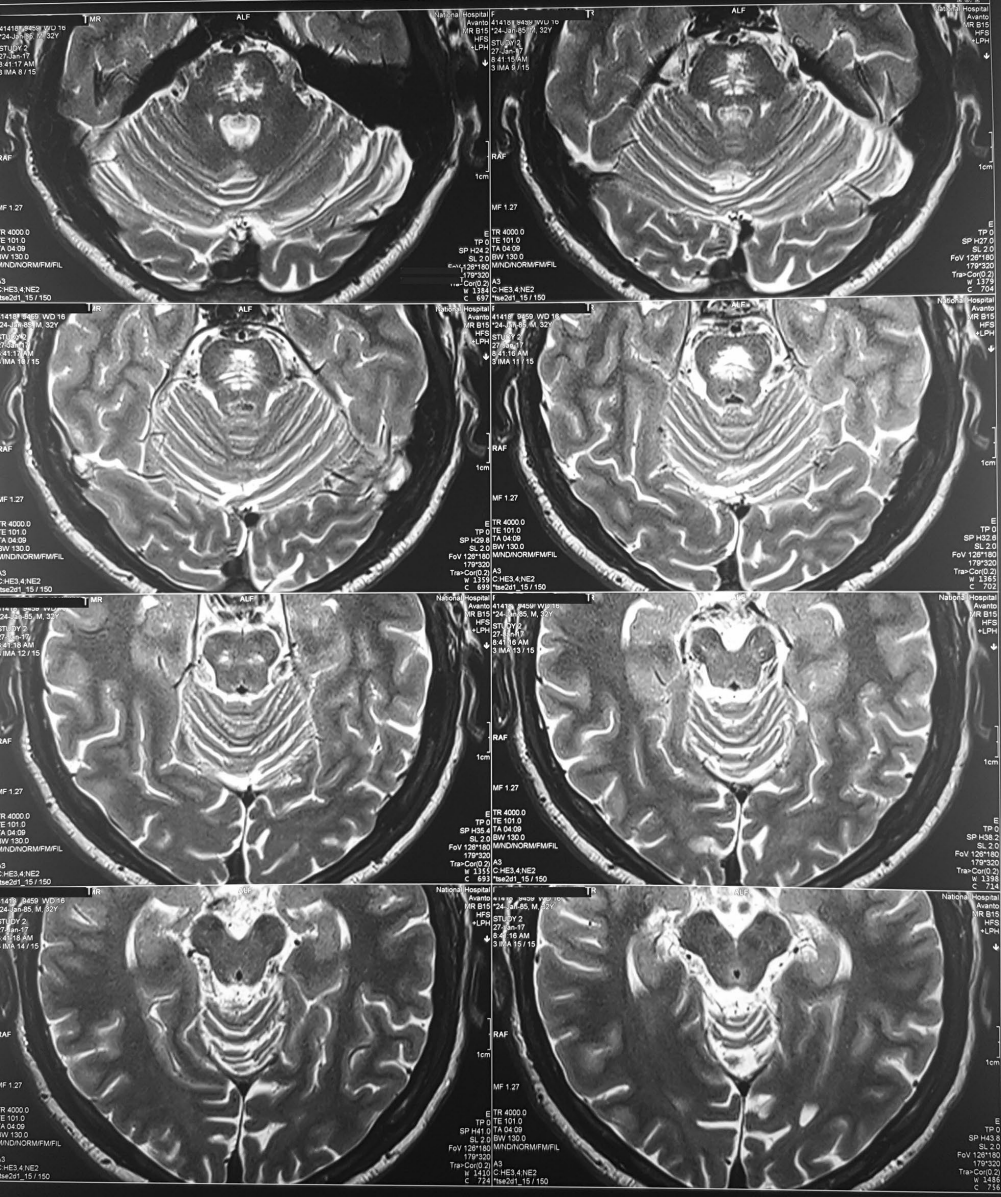
MRI of the brain stem, done 1 month after the first MRI showing signal intensities in FLAIR localized to the pons

**Fig. 4:**
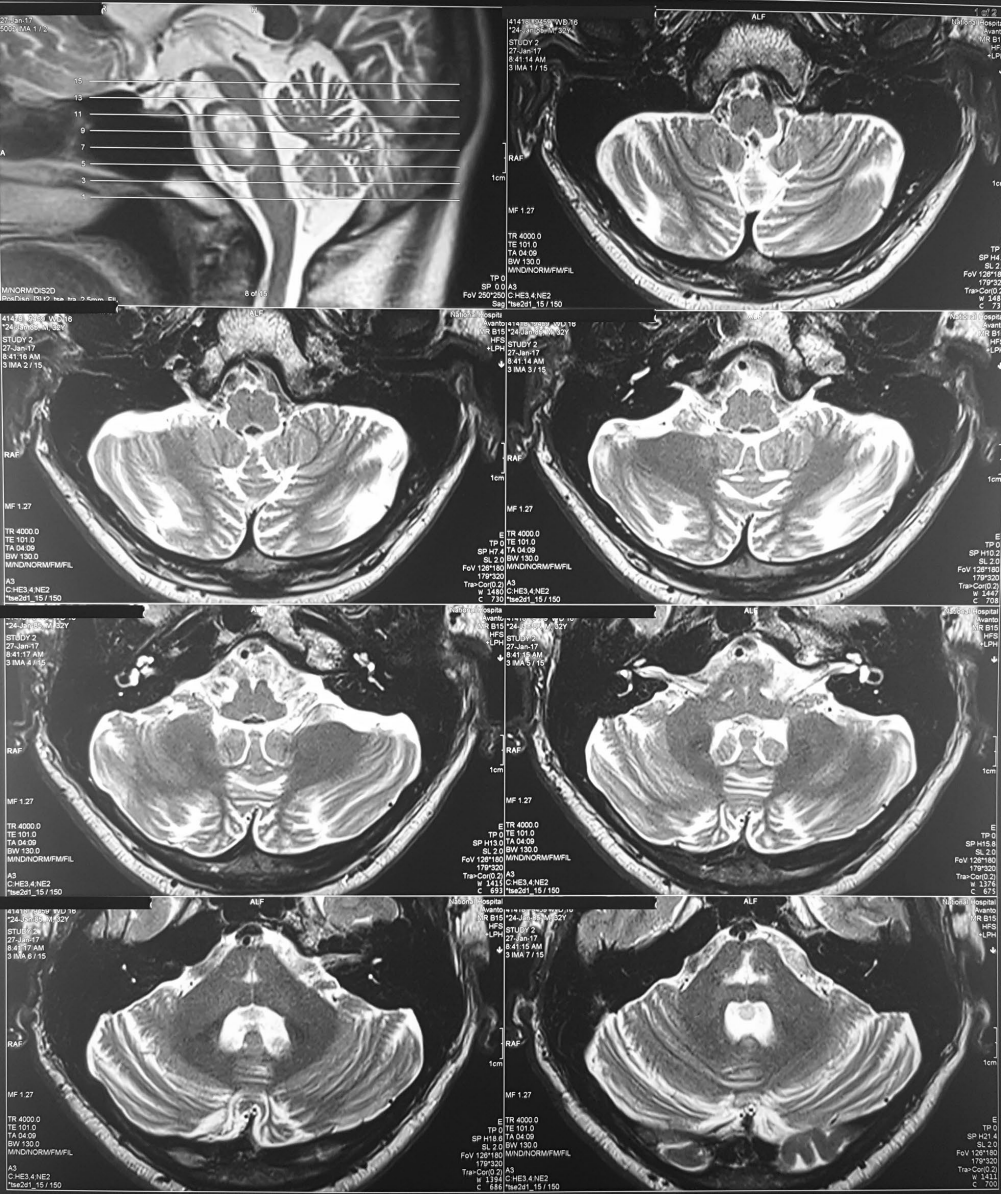
MRI of the brain stem, done 1 month after the first MRI showing normal medulla and hypoglossal nuclei

**Fig. 5:**
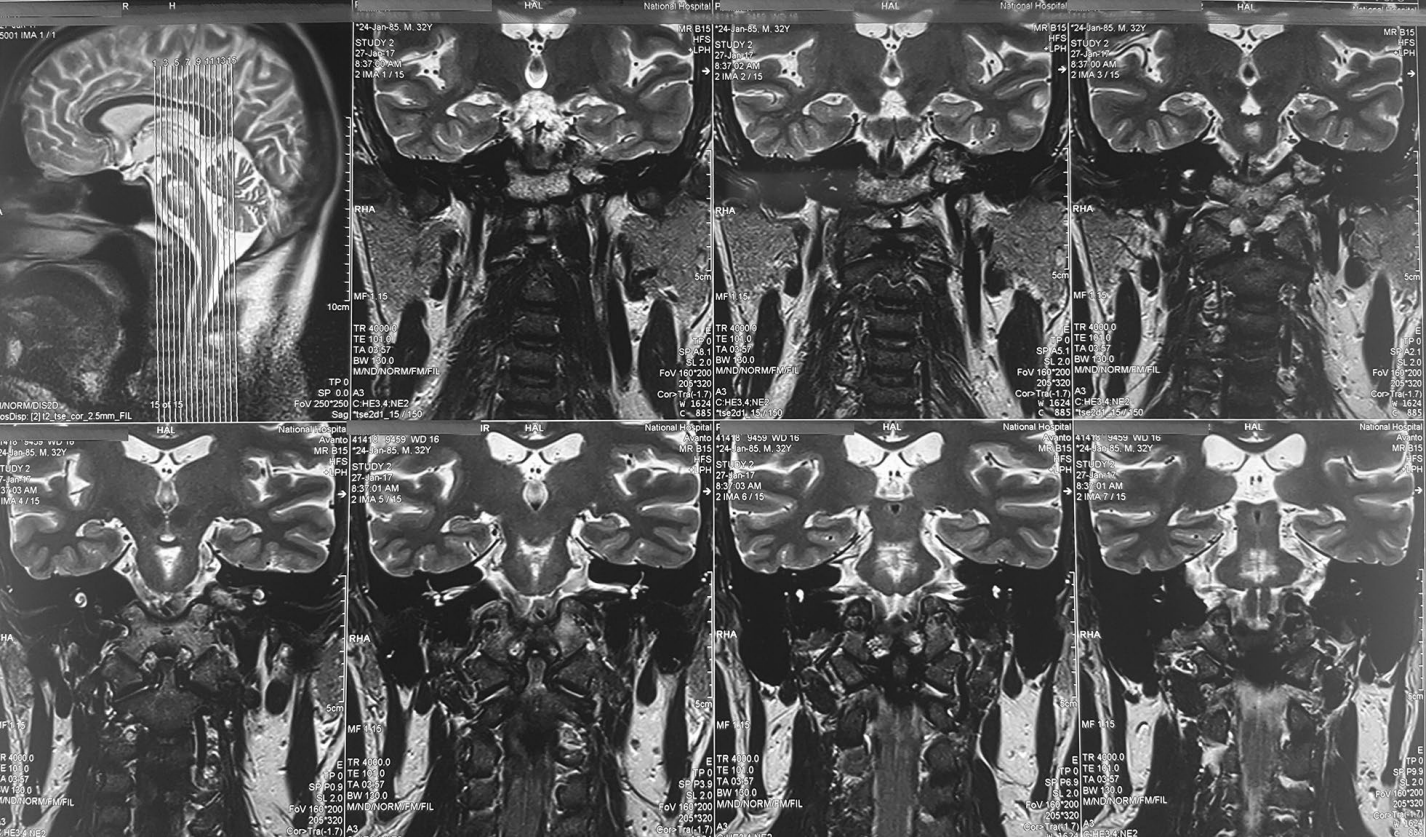
MRI of the brain stem, done 1 month after the first MRI coronal views, showing high signal intensities localized to the pons and not extending into the medulla

Because of the extrapyramidal signs he was started on syndopa (37.5 mg three times a day) and he gradually recovered. Level of consciousness, speech and gait became normal within 1 week. Mini mental score examination done at this stage was normal (30/30). During the follow up, 2 weeks later, bilateral tongue fasciculations without obvious wasting were noted during this time. (Additional file 1: Movie 1) Rest of the cranial nerves were normal. Even though the upper limb and lower limb reflexes were exaggerated, there was no wasting, fasciculations or weakness. He did not have papilledema, down beat nystagmus or mirroring of movements suggesting a lesion at the level of foramen magnum. His sensory system examination and cerebellar examination were normal. EMG showed denervation changes (fibrillations, large amplitude, long duration, complex motor unit action potentials and a reduced interference pattern) in the genioglossal muscle suggesting lower motor type hypoglossal nerve injury. EMG and nerve conduction studies (NCS) of the limbs were normal. MRI was repeated 1 month after the first MRI and showed same changes (T1 low and T2/FLAIR high intensity without diffusion restriction or contrast enhancement) in the central pons (Fig. 2) but previously noted changes in the basal ganglia were not visualized. At the same time we did high resolution MRI of the brain stem and in this, the abnormal signal intensities were localized to the pons (Fig. 3) not extending into the medulla (Figs. 4, 5) and the hypoglossal nuclei and bilateral hypoglossal nerves were normal.

His thyroid function tests, fasting blood sugar, HbA1c, lipid profile, chest X-ray and ultrasound scan of the abdomen were normal. Human immuno deficiency virus 1 and two antibodies, Venereal Disease Research Laboratory, hepatitis B surface antigen, hepatitis C antibody and Anti nuclear antibody were negative. Serum ceruloplasmin level was normal and slit lamp examination did not reveal Kayser–Fleischer rings. We were unable to arrange a total body CT scan to look for occult malignancies that can be missed on the chest X-ray and abdomen ultra sound scan and during the follow up he did not manifest any symptoms or signs of such.

## Discussion and conclusion

Our patient had typical features of ODS such as reduced speech, dysarthria, dysphagia, behavioral disturbances, lethargy, confusion and disorientation. Hyperreflexia is a typical feature of involvement of the upper motor neurons or the corticospinal tracts within the pons. Parkinson like features can be explained by extra pontine osmotic demyelination of the basal ganglia [25]. The current standard method for the diagnosis of osmotic demyelination syndrome (ODS) is brain MRI and is very sensitive for detecting myelinolysis [10]. Pontine demyelination remains the hallmark of the disease [10]. Our patient had the typical MRI findings [10, 26] of ODS and so the clinical and radiological evidence was supportive of our diagnosis of ODS. Even though our patient did not have a documented hyponatremia, it is not a must in ODS/CPM [4, 5, 8, 27–32]. In a systematic review of studies on central pontine and extra-pontine myelinolysis around one quarter of patients with ODS/CPM had sodium of more than 135 mmol/L [10]. Even though our patient’s sodium was normal, he might have had low sodium in the past with ingestion of large quantities of beer which is termed as ‘beer potomania’ [33]. In this patient the management was mainly symptomatic and we started syndopa for extrapyramidal symptoms.

Isolated cranial nerve palsy is rare in CPM. Isolated left sixth nerve palsy was reported in a 30-year-old primigravida with CPM and in this case the MRI lesion correlated anatomically to the location of sixth nerve fascicle [34]. The most interesting clinical manifestation in our patient is the tongue fasciculation with denervation pattern in EMG. Only a few cases of this manifestation have been reported so far. One case report describes a 46-year-old man with a history of alcohol abuse who presented with alcohol withdrawal and developed CPM. He had tongue fasciculations with a deviation of the tongue to the left on protrusion [24]. In this case it was postulated that the presence of tongue fasciculations and deviation to suggest dysfunction at the level of the hypoglossal motor nucleus in the medulla or more peripherally warranting further work-up.

The hypoglossal nuclei are located in the median eminence of the dorsal medulla close to the dorsal motor nucleus of the Vagus nerve and the nucleus tractus solitarius. In our patient the MRI (1.5 Tesla) did not show any lesion in the medulla that could involve hypoglossal nucleus. However, radiologically undetected myelinolysis is possible in the medulla and bilateral hypoglossal nerves, which might have been seen with a stronger strength magnet.

The fibers that arise from the hypoglossal nuclei travel lateral to the medial lemniscus and medial to the inferior olivary nucleus, run in the sulcus between the pyramids and olives, then enter the hypoglossal canal and emerge extracranially. It lies medial and posterior to the internal carotid artery in the carotid sheath and crosses the transverse process of the first vertebral body. Then it runs anteriorly and innervates the tongue muscles. Damage to both hypoglossal nerves will produce tongue fasciculations and atrophy due to denervation. This was reported in cases with brain stem metastasis [19, 21], post traumatic occipital condyle fracture [35, 36], traction of the nerves in vertical subluxation of the odontoid process [37], in head injury [18, 20], following the use of the laryngeal mask airway [23, 38] and post carotid endarterectomy [17, 39]. In our patient we could not find any lesion that can damage the hypoglossal nerve in MRI. Tongue fasciculations are also seen in benign fasciculation syndrome, amyotrophic lateral sclerosis, poliomyelitis, progressive bulbar palsy, spinal muscular atrophy, paraneoplastic syndromes. Acetylcholinesterase inhibitors, benzodiazepine withdrawal, stimulants, organophosphate poisoning, magnesium deficiency and myasthenia gravis [40], but these were unlikely in our patient.

Long-term exposure to methanol, toluene, carbon disulfide, and n-hexane can lead to central nervous system damage, pyramidal signs, parkinsonism and fasciculations [41, 42]. In one patient NCS and EMG had shown fasciculations and low amplitude waves suggestive of polyneuropathy [41]. One case report described a 34 year old floor-layer who developed motor neuron disease with fasciculations and EMG evidence, after being accidentally exposed to a solvent mixture containing methanol and other substances [43]. In one survivor following methanol poisoning extensive denervation was seen in Electromyography suggestive of anterior horn cell involvement [42]. Gille et al. described a patient with chronic motor neuropathy resembling a pseudopolyneuritic type of ALS as a sequel of methanol poisoning [44]. Bourrat et al. reported a woman after voluntary intoxication with methanol who had electromyographic signs of neurogenic atrophy [45]. These reports suggest that methanol can affect the lower motor neurons.

Similar to ODS, in methanol intoxication also MRI will show involvement of basal ganglia, specially putamen and caudate, and pontine tegmentum [46–48]. In such cases, bilateral putaminal lesions appeared hyperintense on *T*2 weighted images and hypointense on *T*1 weighted images [48]. Methanol poisoning can occur with illicit distillation or occult substitution for ethanol, which is possible in our patient. Most of the patients who presented with ODS with normal sodium levels were alcoholic as was this case [5, 8, 9, 32]. Therefore methanol and other toxic substances in alcohol might have played a role in these presentations since clinical manifestations and MRI findings of these poisoning can mimic ODS. Mast et al. also stated that iatrogenic sodium restoration may not be the putative mechanism in all cases of CPM [8] so other possible pathology may coexist. In our patient we were unable to do the toxicology screen and methanol levels on admission because of financial difficulties. But he did not have evidence of optic nerve involvement in MRI or visual evoke potentials, which is seen in methanol poisoning.

In conclusion, tongue fasciculations with denervation of tongue muscles are rarely reported in patients with ODS/CPM. We were unable to explain the exact mechanism for this, but one possibility is radiologically undetected myelinolysis of the medulla. Methanol or other toxic substances that can contaminate alcohol damaging the hypoglossal nerve and causing lower motor type denervation is the other possible mechanism.

## Additional file

**Additional file 1: Movie 1.** Bilateral tongue fasciculations. Description of data: Bilateral tongue fasciculations without obvious wasting were noted during the follow up.

## Abbreviations

ODS: osmotic demyelination syndrome
CPM: central pontine myelinolysis
EMG: electromyogram
MRI: magnetic resonance imaging
FLAIR: fluid-attenuated inversion recovery
CSF: cerebrospinal fluid
NCS: nerve conduction studies
